# Gene drive-mediated population elimination for biodiversity conservation. When you come to a fork in the road, take it

**DOI:** 10.1073/pnas.2218020119

**Published:** 2022-12-13

**Authors:** Bruce A. Hay, Ming Guo

**Affiliations:** ^a^Division of Biology and Biological Engineering, California Institute of Technology, Pasadena, CA 91125; ^b^Department of Neurology, UCLA David Geffen School of Medicine, University of California, Los Angeles, CA 90095; ^c^Department of Molecular and Medical Pharmacology, UCLA David Geffen School of Medicine, University of California, Los Angeles, CA 90095

**Fig. 1.**
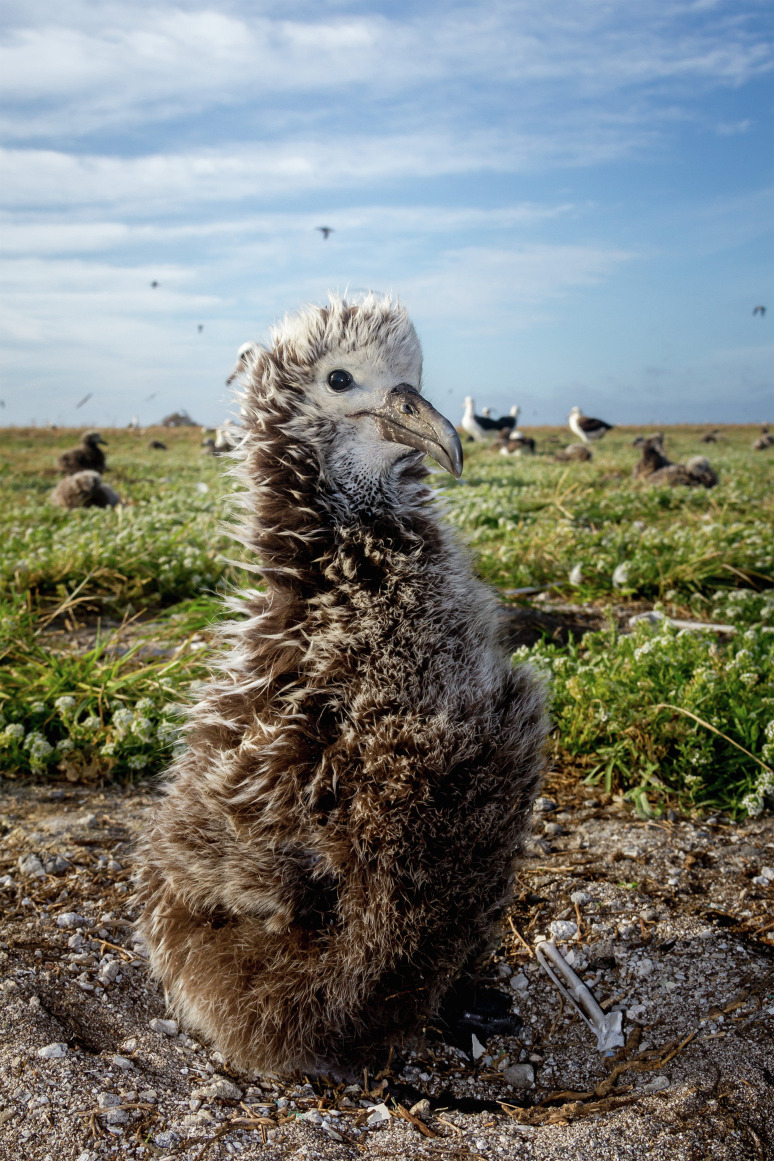
A Laysan albatross chick on Midway Atoll, prey for invasive mice. (credit; Wesley Jolley, Island Conservation).

Gene drive occurs when alleles of genes, multigene cassettes, or large chromosomal regions are transmitted to fertile progeny at greater-than-Mendelian frequencies (50%). Gene drive can be used to bring about population suppression or elimination when the rate at which the drive element increases in frequency outpaces a fitness cost induced by its presence, and the population is driven to an unfit state. Much work has focused on applications involving mosquito vectors of human disease (1). Many other applications have their origin in the global problem of invasive species (2), and thinking about how to ameliorate the many harms they are associated with: food insecurity, human disease, economic loss, environmental degradation, and loss of biodiversity. Invasive species are a major driver of species extinction (3), and island endemic populations are particularly hard-hit. While islands constitute only 6.7% of land area, they host 20% of species and 50% of threatened species and account for 75% of known extinctions since the European expansion (4). Mice and rats are a common culprit (Fig. 1) The primary method for eliminating them utilizes rodenticides. This approach can succeed (5), but the economics and logistics do not scale well with island size. Toxicants can also result in off-target effects on other species, which often precludes their use on islands inhabited by humans and livestock/companion animals. Gene drive–based population suppression provides a solution that eliminates toxicant-based harms and is more humane. It is also species specific and in principle lower cost because it is self-sustaining and takes advantage of the invaders’ tendency to seek out conspecifics even in complex and remote environments. Conversely, a gene drive element must also be unable to bring about suppression in nontarget areas if some individuals manage to “jump ship.” The use of gene drive for population suppression thus involves a fundamental tension between the goals of robust spread and confinement of the desired outcome to the target area. Because islands are isolated, they have been a major focus of research into contexts in which gene drive for population suppression could be tested, to real conservation benefit, while limiting the possibilities for effects elsewhere. The organization GBIRd (https://www.geneticbiocontrol.org) provides an important forum for consideration of these ideas. Work by Gierus, Birand, and colleagues addresses both issues (6). It outlines a method by which island populations of mice (but not other rodents) could be eliminated through gene drive. Importantly, the designs involved contain features that ensure the drive element cannot bring about population suppression in a nontarget population.

“Work by Gierus, Birand and colleagues outlines a method by which island populations of mice (but not other rodents) could be eliminated through gene drive.”

Population elimination using gene drive has been achieved in laboratory cage populations of *Anopheles* mosquitoes (7) using an approach adapted from the behavior of naturally occurring selfish genetic elements known as homing endonuclease genes (HEGs) (8). HEGs increase in frequency by copying themselves from one of a pair of homologous chromosomes to the other using homologous recombination, following creation of a double-strand DNA break on the wild-type homologue. The ability of a synthetic HEG to eliminate a mosquito population depends on homing rates being very high, ~95% or higher. When homing rates are more modest, modeling predicts an internal equilibrium in which the rate of spread is balanced by the fitness cost to carriers, leaving a substantial remaining population (9). Unfortunately, germ line homing rates measured in mice are (to date) too low to be of use (10–12). Thus, gene drive–based approaches to population suppression in mice must take a different path. Nature has provided an interesting alternative in the form of a powerful, although quirky, naturally occurring drive element, the *t* haplotype.

The *t* haplotype is a male meiotic drive element (also known as a segregation distorter; reviewed in ref. 13) studied for almost 100 y. It spans over 40 Mb on chromosome 17 (1.5% of the mouse genome) but is inherited as a unit due to the presence of multiple inversions, which suppress recombination. When present in heterozygous males, sperm that lack the *t* haplotype are disabled, resulting in transmission of *t* to progeny males and females at rates that can exceed 95% depending on the specific *t* allele. Transmission through the female germ line is Mendelian. Multiple genes have been identified as contributors to this behavior (13). However, not enough is known for *t*-like drive to be reverse engineered in mice or other species. In short, the *t* haplotype is a found object, not well understood, but available for use as a gene drive tool.

Early modeling suggested that *t* alleles like *t^w2^*, which are viable and fertile as homozygous females but sterile as homozygous males, might be able to drive small populations of mice to extinction through the creation of populations in which all males are sterile *t* homozygotes. However, *t^w2^* and other *t* alleles are found in nature at modest frequencies, indicating that things are not so simple. Recent work shows that polyandry (mating of females with multiple males) in wild mice is high, and *t*-bearing sperm in *t*/+ heterozygotes (+ indicates wild-type chromosome 17) have decreased competitiveness in comparison with +/+ (14). These forces, and homozygous male sterility, antagonize spread of alleles such as *t^w2^* to high frequency under many conditions (6).

How can the ability of *t^w2^* to spread at super-Mendelian frequencies be utilized even if it is unable to directly drive the population to an unfit state? Gierus, Birand, and colleagues proposed placing Cas9 and a gRNA at a neutral position within the *t* haplotype. In this hybrid gene drive element, which they refer to as *t*_CRISPR_, Cas9 and the gRNA cleave and (hopefully) create loss-of-function (LOF) alleles in the male germ line of the prolactin (*Prl*) gene, which is required for female fertility. The goal with *t*_CRISPR_ is for *t*-based segregation distortion in males to pump the Cas9/gRNAs cassette to high frequency within the population. The latter, through cleavage followed by inaccurate repair in males, will continuously produce LOF alleles at the independently segregating *Prl* locus. The hope is that the combination of *t*-based drive and accumulation of *Prl* LOF alleles will drive the population to an unfit state that contains a high frequency of infertile homozygous *Prl* mutant females along with some frequency of infertile homozygous *t* males. The combination of these two effects, they propose, could eliminate populations under a wider range of parameters than with *t^w2^* alone.

Modeling of *t*_CRISPR_ in spatially explicit populations supports this idea (6). *t*_CRISPR_ is also predicted to work well with levels of cleavage and LOF allele creation (~80%) that are plausibly achieved. As with other Cas9-based methods for population suppression, *t*_CRISPR_ can fail if resistant alleles appear. These are alleles, either naturally occurring or generated by inaccurate repair following cleavage, that are now uncleavable (because the gRNA no longer base pairs completely with the target) but still allow for the synthesis of a functional product. They are rapidly selected for when in competition with low-fitness LOF alleles. Their appearance can be prevented or at least delayed by targeting the fertility gene at multiple positions.

Modeling also identifies an important set of interactions between *t^w2^* and *t*_CRISPR_ when both are present in the same population. Under these conditions, *t*_CRISPR_ is at a disadvantage as compared with *t^w2^* and is eventually lost from the population. This happens because *t*_CRISPR_ alleles—which are continuously generating *Prl* LOF alleles in the male germ line—find themselves in dead-end homozygous LOF prolactin female progeny more often than do *t^w2^* alleles. An important implication of this dynamic noted by the authors is that if Cas9 or gRNAs mutate to inactivity, which will inevitably happen if population extinction does not occur, the *t*_CRISPR_ allele will be converted (functionally) to a *t^w2^* allele. Once this happens (or migration of *t^w2^*-bearing individuals to the island occurs), *t*_CRISPR_ may lose its advantage. This will occur even if new versions of Cas9/gRNAs targeting a different fertility gene are introduced into the *t^w2^* haplotype—because they would still be competing with existing *t^w2^*. Multiplexing of Cas9 and the gRNAs to bring about redundancy can forestall but not prevent this process. In short, with a *t*_CRISPR_-based approach, it is important to get population elimination right the first time as there will not be a second chance once significant levels of *t^w2^* are present in the population. This same modeling result also has, however, the important positive corollary that movement of some *t*_CRISPR_ individuals to a larger mainland will never result in population suppression because *t^w2^* alleles, which we know exist in a harmonious balance with wild type in the wild, will inevitably arise from *t*_CRISPR_ in large populations.

The authors test the *t*_CRISPR_ idea using a format in which a gRNA targeting the *Prl* gene is inserted into the *t^w2^* haplotype, and Cas9, expressed under the control of a male germ line–specific promoter, sits elsewhere. In a nutshell, when males carry both constructs, segregation distortion is maintained (95%), and indel creation (and thus hopefully LOF allele creation) in *Prl* occurred in the male germ line at a respectable frequency of 80%. Finally, analysis of previously published pooled whole genome sequences from multiple islands (15) shows that *t* alleles are often, although not always, absent.

These modeling and experimental results argue that *t*_CRISPR_ provides a path to elimination of invasive populations of mice on some islands. The fact that *t*_CRISPR_ will inevitably break down to *t^w2^* also makes it biologically implausible that t_CRISPR_ could achieve a similar end on a mainland. Related to this last point, the authors note that deliberate introduction of *t^w2^* into a neighboring mainland of concern would (if it is not already there) serve a similar blocking function. A conceptually important additional method for limiting suppression to a target island, that applies to all DNA sequence modification-based drives, focuses on targeting what are known as locally fixed alleles—alleles that are fixed (present on both alleles of all individuals in the population) on the target island but not on the mainland (16). In the context of *t*_CRISPR_, alleles of a gene required for female fertility that are fixed on the island but not on the mainland would be targeted. Mainland alleles different from the fixed island alleles are (because they are not recognized by the gRNAs used on the island) functionally resistant alleles. Modeling shows that even a low frequency of such alleles will prevent population suppression (16). Sequence analysis has begun to identify target genes for the locally fixed allele approach (15).

Where do we go from here? Multiple unknowns remain. In particular, the life history of mice on target islands should be explored to provide values for the many variables that can influence drive outcome. Any *t*_CRISPR_ strain to be used will also need to be backcrossed extensively into the target population genetic background to maximize fitness. All this characterization is necessary so as not to end up in a situation in which a *t*_CRISPR_ is released on an island, only to find some years later that it is not quite good enough to bring about eradication, leaving the island immune to further *t*-based suppression strategies.

Finally, a few words about time. Gene drive is sometimes portrayed in the popular press as a tool that will scythe its way through a population like a hot knife through butter, rapidly bringing about population elimination. This is not the case. Modeling by Gierus, Birand, and colleagues estimates times to eradication of ~20 to 30+ y depending on the values for the variables noted above (and of course the *t*_CRISPR_ introduction frequency). Thus, if a gene drive like *t*_CRISPR_ is introduced onto an island, there will be ample time to monitor and learn from its dynamics. A real question is if endangered species on some of these islands will be able to hold on long enough to be the beneficiaries of this technology.
